# Treatment of Advanced Glaucoma Study: a multicentre randomised controlled trial comparing primary medical treatment with primary trabeculectomy for people with newly diagnosed advanced glaucoma—study protocol

**DOI:** 10.1136/bjophthalmol-2017-310902

**Published:** 2017-10-26

**Authors:** Anthony J King, Gordon Fernie, Augusto Azuara-Blanco, Jennifer M Burr, Ted Garway-Heath, John M Sparrow, Luke Vale, Jemma Hudson, Graeme MacLennan, Alison McDonald, Keith Barton, John Norrie

**Affiliations:** 1 Department of Ophthalmology, Nottingham University Hospitals NHS Trust, Nottingham, UK; 2 Centre for Healthcare Randomised Trials (CHaRT), Health Services Research Unit, University of Aberdeen, Health Services Research Unit, University of Aberdeen, Aberdeen, UK; 3 Centre for Public Health, Queen’s University Belfast, Royal Victoria Hospital, Belfast, Northern Ireland, UK; 4 School of Medicine, University of St Andrews, St Andrews, Fife, UK; 5 Institute of Ophthalmology, University College London, London, UK; 6 Moorfields Eye Hospital, NHS Foundation Trust, London, UK; 7 Department of Ophthalmology, Bristol Eye Hospital, Bristol, UK; 8 Institute of Health and Society, Newcastle University, Newcastle, UK

**Keywords:** glaucoma, treatment medical, intraocular pressure, clinical trial, treatment surgery

## Abstract

**Background:**

Presentation with advanced glaucoma is the major risk factor for lifetime blindness. Effective intervention at diagnosis is expected to minimise risk of further visual loss in this group of patients.

**Aim:**

To compare clinical and cost-effectiveness of primary medical management compared with primary surgery for people presenting with advanced open-angle glaucoma (OAG).

**Methods:**

*Design*: A prospective, pragmatic multicentre randomised controlled trial (RCT).

**Setting:**

Twenty-seven UK hospital eye services.

**Participants:**

Four hundred and forty patients presenting with advanced OAG, according to the Hodapp-Parish-Anderson classification of visual field loss.

**Intervention:**

Participants will be randomised to medical treatment or augmented trabeculectomy (1:1 allocation minimised by centre and presence of advanced disease in both eyes).

**Main outcome measures:**

The primary outcome is vision-related quality of life measured by the National Eye Institute—Visual Function Questionnaire-25 at 24 months. Secondary outcomes include generic EQ-5D-5L, Health Utility Index-3 and glaucoma-related health status (Glaucoma Utility Index), patient experience, visual field measured by mean deviation value, logarithm of the mean angle of resolution visual acuity, intraocular pressure, adverse events, standards for driving and eligibility for blind certification. Incremental cost per quality-adjusted life-year (QALY) based on EQ-5D-5L and glaucoma profile instrument will be estimated.

**Results:**

The study will report the comparative effectiveness and cost-effectiveness of medical treatment against augmented trabeculectomy in patients presenting with advanced glaucoma in terms of patient-reported health and visual function, clinical outcomes and incremental cost per QALY at 2 years.

**Conclusions:**

Treatment of Advanced Glaucoma Study will be the first RCT reporting outcomes from the perspective of those with advanced glaucoma.

**Trial registration number:**

ISRCTN56878850, Pre-results.

## Introduction

Advanced visual field (VF) loss at glaucoma diagnosis is the major risk factor for lifetime blindness.^1^ Reducing intraocular pressure (IOP) by medications, surgery or laser is the only currently available treatment option for glaucoma.^2–9^ Glaucoma guidelines suggest that primary surgery may be a suitable option for presentation with advanced glaucoma.^10–12^

Evidence from a systematic review,^2^ including four randomised controlled trials (RCTs),^3 13–15^ comparing primary medical versus surgical treatment for open-angle glaucoma (OAG) suggests that treatment failure was more likely when the primary treatment was medication (OR 3.90, 95% CI 1.60 to 9.53; HR 7.27, 95% CI 2.23 to 25.71). Data from the largest and most recent of these RCTs found no substantial difference in patient-reported outcomes between intervention groups at 5 years.^16^ However, this study did not include people with advanced glaucoma. Subsequent to these studies being completed, many more glaucoma medications have become available with better efficacy in terms of IOP lowering and less side effects.^17^ Trabeculectomy remains the conventional primary surgery, but the technique has evolved^18 19^ to include the use of wide application mitomycin C (MMC),^20^ releasable sutures^21^ and extensive postoperative manipulation^22^ to improve outcomes.^23^

Given that advanced glaucoma is a sight-threatening disease with uncertainty about the best primary treatment option, there is a need for a high-quality study comparing contemporary medications with primary trabeculectomy for newly diagnosed advanced glaucoma reporting outcomes relevant to patients, clinicians and health services.

## Patients and methods

The Treatment of Advanced Glaucoma Study (TAGS) is a pragmatic^24 25^ multicentre RCT comparing primary medical treatment with primary augmented trabeculectomy with the primary outcome assessment at 2 years post randomisation. Participants will be randomised to medical treatment or augmented trabeculectomy (1:1 allocation minimised by centre and presence of advanced disease in both eyes). The trial’s protocol reflects routine care to ensure that the results are representative of current clinical practice.

### Participants

Adults (≥18 years) presenting with advanced (severe) glaucoma in at least one eye will be invited to participate. Advanced disease is classified according to the ‘severe’ category of VF loss using the Hodapp classification of glaucoma severity^26^ (see box for a full list of eligibility criteria). Two baseline Humphrey VFs (24-2 SITA Standard) will be performed on all participants prior to randomisation to confirm eligibility. If both eyes are eligible, it is the participant, not the eye, who is randomised to treatment; both eyes receive the same intervention.

Box **Inclusion and exclusion criteria**

**Inclusion criteria**

1. Severe glaucomatous visual field loss (Hodapp classification) in one or both eyes at presentation on any of these criteria:
  - Mean deviation <−12.00 dB.
  - >50% of points defective in the pattern deviation probability plot at the 5% level (>27 points on 24-2 HVF).
  - >20 points defective at the 1% level.
  - A point in the central 5 degrees has a sensitivity of 0 dB.
  - Points within 5 degrees of fixation<15 dB sensitivity in both upper and lower hemifields.
2. Diagnosis of open-angle glaucoma including pigment dispersion glaucoma, pseudo-exfoliative glaucoma and normal tension glaucoma.
3. Adult≥18 years.
4. Ability to provide informed consent and willingness to participate in the trial.

**Exclusion criteria**

1. Visual field defects not meeting advanced visual field loss criteria in either eye.
2. Primary angle closure glaucoma and all other secondary glaucomas.
3. Inability to undergo incisional surgery due to inability to lie flat or unsuitable for anaesthetic.
4. High risk of trabeculectomy failure such as previous conjunctival surgery, complicated cataract surgery.
5. Women who are
  - (i) pregnant, (ii) nursing, (iii) planning a pregnancy and (iv) of childbearing potential not using a reliable method of contraception.

Participants will be recruited from an estimated 27 hospitals in the UK over a 36-month period. Participants will be followed for 2 years from randomisation (see table 1 for the schedule of assessments).

**Table 1:** Timing of outcome measurements

	Baseline	Post randomisation (months)						
		1	3	4	6	12	18	24
*Clinical*								
Medical history	x							
Humphrey visual field mean deviation	x			x		x		x
Esterman Visual Field	x							x
LogMAR visual acuity	x			x		x		x
Intraocular pressure	x			x		x		x
Standard clinical examination	x					x		x
*Patient experience*								
NEI-VFQ-25	x			x		x		x
EQ-5D-5L*	x	x	x		x	x	x	x
HUI-3*	x	x	x		x	x	x	x
GUI*	x	x	x		x	x	x	x
Patient experience questions	x	x	x		x	x	x	x
*Health economics*								
Healthcare utilisation (including hospital visits)				x		x		x
Participant cost				x		x		x
Participant time and travel							x	

*Additional questionnaire undertaken immediately prior to trabeculectomy surgery; discrete choice experiment at 27 months;

GUI, Glaucoma Utility Index; HUI-3, Health Utility Index; LogMAR, logarithm of the mean angle of resolution; NEI-VFQ25, National Eye Institute Visual Function Questionnaire (25 items).

### Study interventions

An individualised target IOP will be set at baseline according to the algorithm developed by the Canadian Consensus on target IOP setting.^27^

In the primary medical treatment arm, participants will start on one or more medications at their initial visit depending on the judgement of the treating clinician and as advised by the National Institute for Health and Care Excellence (NICE) glaucoma guideline,^10^ with subsequent additional medication based on clinician judgement. All currently available licensed drops may be used. If drops fail to lower the IOP adequately, oral carbonic anhydrase inhibitors may be used. If medical treatment fails, patients will be offered glaucoma surgery.

In the primary trabeculectomy group, surgery will be undertaken within 3 months of randomisation by a surgeon who specialises in glaucoma or a glaucoma fellow who has performed at least 30 trabeculectomies. Patients’ IOP will be medically controlled until glaucoma surgery is undertaken. In trabeculectomy augmented with MMC, a ‘guarded fistula’ is created by making a small hole in the eye, covered by a flap of partial thickness sclera.^18 28^ After glaucoma surgery, medical treatment may be introduced if the IOP is above the desired target.

Where both eyes are eligible and the participant is allocated to the surgery arm, a decision as to which eye will undergo trabeculectomy first will be made by the clinician and patient together. A period of 2–3 months will be allowed between these operations.

### Summary of outcomes

The primary outcome is vision-related quality of life (QoL) collected with the National Eye Institute Visual Function Questionnaire (25 items) (NEI-VFQ25). Secondary outcomes include clinical outcomes: mean deviation (MD) on Humphrey VF testing, logarithm of the mean angle of resolution (logMAR) visual acuity, IOP, complications of treatment, need for cataract surgery; patient-reported outcomes: generic health status (EQ-5D-5L and Health Utility Index (HUI-3)), glaucoma health status (glaucoma profile instrument (GPI)), patient experience; and health economic outcomes: quality-adjusted life-year (QALYs) and incremental cost per QALY (tables 1 and 2).

**Table 2:** Trial outcomes

	Outcome	Analysis
Primary objective	*Patient-centred:* Vision-specific health profile (NEI-VFQ25) at 24 months	Intention to treat.
Secondary objectives	*Patient-centred:* Patient-reported health status, HUI-3; EQ-5D-5L (5-level), GUI, NEI-VFQ25; patient experience. *Clinical:* Visual field mean deviation at 24 months. Intraocular pressure; logMAR visual acuity; need for cataract surgery; visual standards for driving; registered visual impairment; safety. *Economic:* Incremental cost per QALY gained (based on responses to the EQ-5D-5L; HUI-3); incremental cost per QALY gained (based on responses to glaucoma profile instrument (GPI)); incremental costs to the NHS, personal social services and patients.	Profile over time will be analysed by repeated measures using a linear mixed model. Subgroup analyses will explore potential effect modification of gender, age, one or both eyes affected and extent of visual field loss at baseline (<–20 dB, ≥20 dB) on the primary outcomes.

GPI, glaucoma profile instrument; GUI, Glaucoma Utility Index; HUI-3, Health Utility Index; LogMAR, logarithm of the mean angle of resolution; NEI-VFQ25, National Eye Institute Visual Function Questionnaire (25 items); NHS, National Health Service; QALY, quality-adjusted life-year.

The NEI-VFQ25 is a vision-specific patient-reported QoL instrument widely used to evaluate visual outcomes in glaucoma.^29–32^ Generic EQ-5D-5L,^33^ HUI-3^34^ and the glaucoma-specific, GPI^35^ will be collected to generate utility outcomes. VF MD change measures the amount of vision loss due to glaucoma. VF damage is the major clinical measure of the functional impact of glaucoma, which adversely influences QoL.^29 36–38^ VFs eligible for analysis will have to achieve a predefined reliability criterion (false positives<15%). VFs will be assessed by an independent VF reading centre, masked to the treatment received by the study participant. The independent reading centre will evaluate MD change and whether the Esterman Visual Fields achieve driving standard levels.

IOP will be measured by Goldmann tonometry in mm Hg. The measurement will be undertaken by two observers, the first observer making the measurement and the second reading it from the measurement dial. Two measurements will be taken and a third if there is a discrepancy >3 mm Hg between the first two. The mean of these values will be used.

Best-corrected logMAR visual acuity at 4 m will be measured on each eye and binocularly. Complications of surgery, need for cataract surgery and therapy changes, will be captured from the participants’ case records. All clinical outcomes will be recorded on a trial-specific case report form (CRF). If a participant is eligible to be registered as visually impaired or severely visually impaired in the opinion of the consulting clinician, this will be recorded in the study CRF at 24 months.

### Economic outcomes

Costs of treatments (surgery/medications) including time in hospital and secondary care use will be based on data collected in the trial CRFs. Primary care, personal social service use and participant costs will be collected via questionnaires at 4, 12 and 24 months post randomisation. Responses to the EQ-5D-5L (recommended for use by NICE in the UK), HUI-3 (which has vision-related questions) and GPI (which should be more sensitive to changes in glaucoma) will be combined with the relevant scoring tariffs^35 39 40^ to produce QALYs. Glaucoma-specific scoring tariffs will be elicited in this study using a discrete choice experiment, replicating the method adopted by Burr *et al* 35 with a sample of people with advanced glaucoma. Costs and QALYs will be combined in a cost–utility analysis both ‘within trial’ and modelled over the patient’s lifetime; with the model informed by previous models conducted.^41–43^

### Sample size

The primary outcome is patient-reported vision-related QoL measured by the NEI-VFQ25 at 24 months. A study with 190 participants in each group will have 90% power at 5% significance level to detect a difference in means of 0.33 of an SD; this translates to 6 points on the NEI-VFQ25 assuming a common SD of 18 points from previous work in patients with advanced glaucoma.^44^ Seven points is a likely minimally important difference based on our pilot work on NEI-VFQ25 scores in patients with glaucoma,^45^ but there is uncertainty and so we have opted for a more conservative six-point difference, which is supported by the literature for another chronic eye disease, macular degeneration.^46^ Assuming a 13.5% dropout due to death and participants declining follow-up, we need to randomise 440 participants to detect this difference.

For the secondary clinical outcome (VF MD), the study has 90% power at a 5% level of significance to detect a 1.3 dB difference in MD between groups after 2 years. This was derived from a subgroup of patients with advanced glaucoma^2 47^ and is a clinically meaningful difference in the context of advanced glaucoma, given that progressive loss tends to be linear,^48 49^ with small changes over a short period extrapolating to large changes over a patient’s lifetime.

### Recruitment

Patients likely to be eligible for the trial will be identified at the initial consultation for glaucoma and provided with a participant information leaflet. Eligible participants, who agree to take part, will sign a consent form before being randomised. Recruitment will be performed by the ophthalmologist and must be completed within 3 months of glaucoma diagnosis.

Randomisation will be performed at recruitment centres using the remote-automated computer randomisation service at the Centre for Healthcare Randomised Trials, either over the internet or by telephone. Randomisation will be minimised by centre and whether there is advanced glaucoma in both eyes. The unit of randomisation is the participant (not the eye).

### Adverse events/safety reporting

Serious adverse events (SAEs) related to participants’ glaucoma care or participation in the trial will be reported in accordance with the guidance from the UK Health Research Authority (http://www.hra.nhs.uk/research-community/during-your-research-project/safety-reporting/). Table 3 describes the expected adverse events.

**Table 3:** Expected adverse events

Visual acuity loss of vision (any of the following): Irreversible loss of 10 ETDRS letters of logarithm of the mean angle of resolution visual acuity Loss of two or more stages of categorical visual acuity measurement (count fingers, hand motion, light perception, no light perception) Any loss to no light perception	
Intervention 1 Medical treatment*	Intervention 2 Trabeculectomy with mitomycin C
Redness, stinging, itching, transient blurred vision, eyes watering, ocular discomfort, allergy, eyelash growth, change in skin colour around eye, change in iris colour, shortness of breath, unpleasant taste in mouth, dry mouth, fatigue, kidney stones, skin rash, cataract formation and retinal detachment*	Discomfort, blurred vision, corneal epithelial defect, conjunctival button-hole, flap dehiscence, intraocular pressure too low, transient choroidal effusion, suprachoroidal haemorrhage, hyphema, early bleb leak, late bleb leak, shallow anterior chamber (grades 1–3), iris incarceration, persistent uveitis, transient or permanent ptosis, macular oedema, malignant glaucoma, corneal decompensation, cataract formation and retinal detachment, bleb infection, bleb related endophthalmitis, permanent severe loss of vision at time of surgery (<1/500), bleeding in the eye, broad complex tachycardia while under general anaesthetic, postoperative dizziness

*In some case, these symptoms may be due to preservatives in the drops—if this is the case, preservative free drops can be used.

These are based on knowledge of adverse events associated with augmented trabeculectomy and the relevant product information documented in the summary of product characteristics (SmPC). The latest online version of the appropriate SmPC will be considered in the assessment of an adverse event.

All related SAEs will be summarised and reported to the Ethics Committee, the funder and the Trial Steering Committee in regular progress reports. Any serious, related and unexpected events will be expeditiously reported (no later than 15 calendar days after the trial team are first aware of the event).^50^

### Procedure

Table 1 displays the timing of the trial’s outcome measurements. At baseline, following consent but prior to randomisation, participants’ relevant medical history, IOP, Humphrey visual fields and best-corrected logMAR visual acuity will be measured. A general ophthalmic examination including central corneal thickness will also be undertaken. Participants will complete a questionnaire including the NEI-VFQ25, EQ-5D-5L, HUI-3, GPI and a question asking about the patient’s experience of glaucoma.

**Figure 1:**
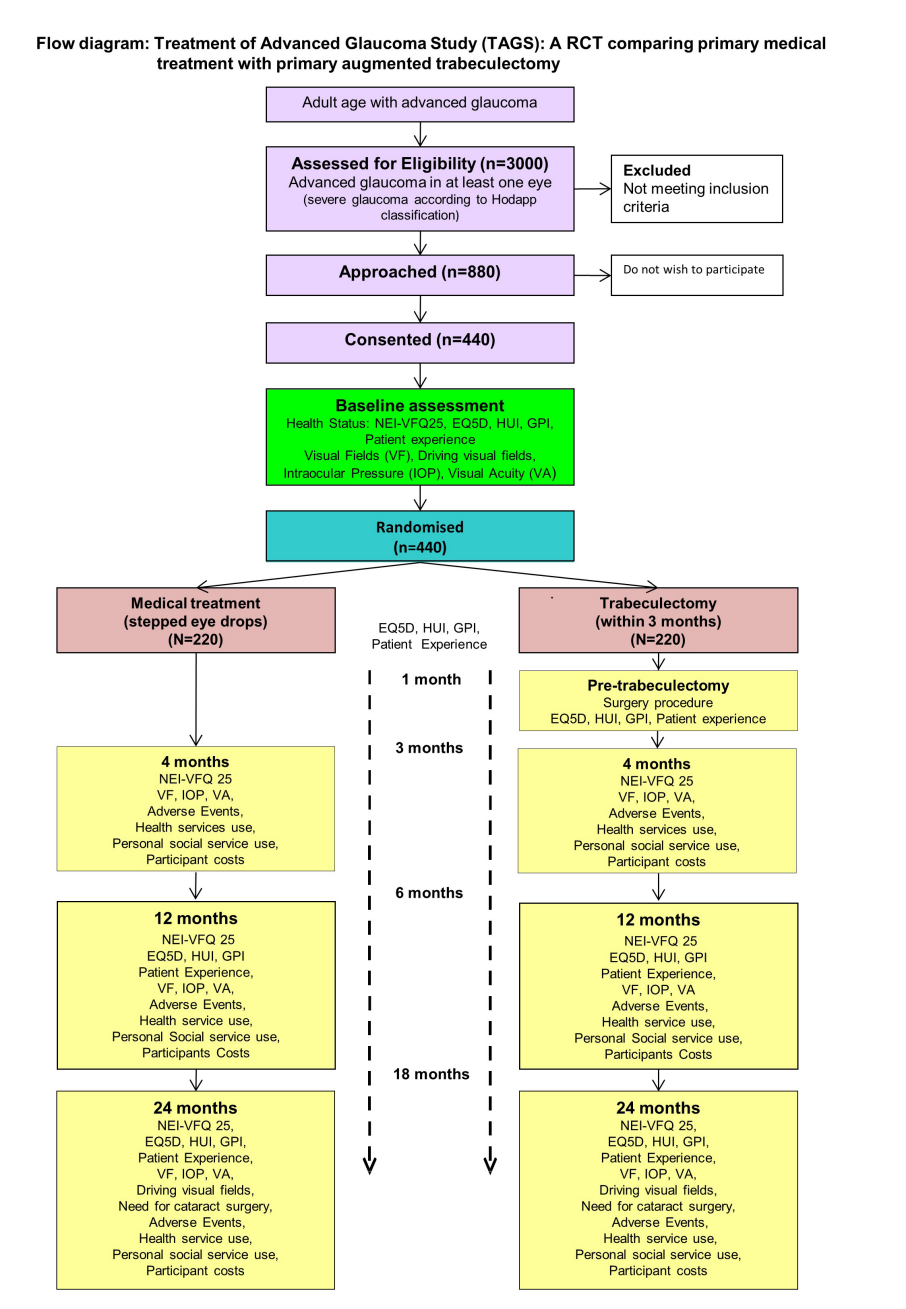
Study flow chart with outcome timeline. GPI, glaucoma profile instrument; HUI, Health Utility Index; NEI-VFQ25, National Eye Institute Visual Function Questionnaire (25 items); RCT, randomised controlled trial.

Most of the outcomes will be gathered at four time points across the 2-year follow-up as illustrated in figure 1 (see table 1 for details).

### Statistical analyses

Baseline characteristics, follow-up measurements and safety data will be described using the appropriate descriptive summary measures. The primary outcome, NEI-VFQ25 score, will be analysed using linear regression adjusting for baseline NEI-VFQ25 score and other prognostic variables, for example, amount of vision loss and IOP at baseline, one or both eyes affected by advanced glaucoma. We will also explore the profile of NEI-VFQ25 score over time using a linear mixed model to analyse data from all time points. All models will include a random effect for surgeon. The primary analysis strategy will be intention to treat. We will describe the amount and patterns of missing data and use appropriate methods,^51^ if required, to run sensitivity analysis to test assumptions.^52^

There is potential for cross-over to the alternative treatment (non-compliance with allocation). In addition to the ‘effectiveness’ estimate from the intention-to-treat analyses, we will explore ‘efficacy’ estimates using causal modelling methods suitable for complex interventions,^53^ if required.

Secondary outcomes will be analysed with a similar strategy, with models suitable for the outcome (ie, logistic regression for the dichotomous outcome ‘need for cataract surgery’ at 2 years). Outcomes measured at the eye level will be analysed initially using data from the index eye only. For participants with both eyes eligible, the eye with the better MD value (less severe VF damage) is nominated the index eye for the purposes of the statistical analyses.

Sensitivity analysis using data from all eligible eyes will be done, including a random effect at the participant level to reflect the lack of independence of eyes within participants. All treatment effects will be derived from these models and presented with 95% CIs.

Planned subgroup analyses are intended to explore potential effect modifications of gender, age, one or both eyes affected and extent of VF loss in the index eye at baseline (<–20 dB, ≥20 dB) on the primary outcomes. Subgroup by treatment interaction will be assessed by including interaction terms in the models outlined above.

The Data Monitoring Committee will monitor safety and other data at 6-monthly intervals during the recruitment phase of the trial. Due to the staggered nature of recruitment and the primary outcome measurement at 2 years, we do not anticipate early termination for benefit. We have planned for one main effectiveness analysis at the end of the trial. All statistical analysis will be detailed in the Statistical Analysis Plan, which will be completed before the final analysis is started.

The study adheres to the tenets of the Declaration of Helsinki and the principles of Good Clinical Practise (GCP), and is in accordance with all applicable regulatory guidance, including, but not limited to, the Research Governance Framework. TAGS’ protocol and patient-facing documentation were prospectively reviewed and approved by the Derby 1 Research Ethics Committee (Ref Number 13/EM/00395). Local NHS Research and Development (R&D) approvals were obtained prior to commencement of the trial at the participating sites. An independent data and safety monitoring committee oversees the trial.

### Funding

The trial is funded by a grant awarded by the Health Technology Assessment (NIHR HTA) programme (project number 12/35/38). TAGS is registered on the ISRCTN registry: 56878850.

Nottingham University Hospitals NHS Trust sponsors the trial and provides the necessary trial insurance.

## Discussion

Major changes in the efficacy, safety and variety of glaucoma drops available over the last two decades as well as significant modifications to trabeculectomy surgery have improved safety and efficacy outcomes.^18 23^

There is uncertainty regarding the optimal primary treatment pathway for people presenting with advanced glaucoma. There is a recognition that advanced glaucoma at presentation needs to be treated more aggressively.^10–12^ In the UK, only about 30% of UK ophthalmologists follow the NICE guidelines for primary surgery.^54^ Clinicians indicated that robust evidence supporting the best primary approach would change their practice.^54^ The TAGS study aims to address this evidence gap.

Patient-reported outcomes are an important component of treatment choice and are related to the extent of VF loss.^50 55^ TAGS is the first glaucoma surgery RCT with patient-reported vision-related QoL as the prespecified primary outcome. As both treatments being tested have proven efficacy in treating glaucoma, there may be little measurable difference in clinical outcomes. Loss of visual function leads to disability in tasks of daily living.^56–64^ In patients with advanced glaucoma, because of their limited visual reserve, further VF loss is likely to result in noticeable difference in visual function in all aspects of life related to vision.

The participant ‘journey’ through both treatment options following diagnosis will be recorded with a vision-specific (NEI-VFQ25), generic (EQ-5D-5L and HUI-3) and glaucoma-specific (GPI) patient-reported questionnaires capturing any QoL differences between a primary surgery and a primary medication care pathway. We will also explore which generic health status questionnaires, one with a specific visual function domain (HUI-3) and one without (EQ-5D-5L), is sensitive to any differences between primary treatment options.

Generic QoL assessments have not been previously undertaken in a medicine versus surgery trial for glaucoma, although the Collaborative Initial Glaucoma Treatment Study^3 31^ reported on differences in local eye symptoms and visual activities related to interventions.^16^

Measuring our outcome at 2 years will allow us to track the patient journey through the active initial management period for their glaucoma and will provide us with meaningful information regarding patient-reported outcomes, clinical and safety outcomes and economic outcomes of the two treatment options tested. However, glaucoma is a slowly progressive condition and it may take many years for differences in the outcomes of treatment choices to be revealed.^47 65–67^ Patients with glaucoma live for many years following their diagnosis^68–72^ and it is therefore essential to obtain further information about the effect of treatment options in the medium to long term to better inform our patients of lifetime outcomes with different treatment options and allow more effective economic modelling. We therefore plan to seek further funding to allow further evaluation of this cohort at 5 years.
